# Short-Chain Sulfur Confined into Nitrogen-Doped Hollow Carbon Nanospheres for High-Capacity Potassium Storage

**DOI:** 10.3390/nano14060550

**Published:** 2024-03-20

**Authors:** Wenhan Liu, Tengfei Shi, Fang Liu, Chen Yang, Fan Qiao, Kang Han, Chunhua Han, Jiashen Meng, Xuanpeng Wang

**Affiliations:** 1 State Key Laboratory of Advanced Technology for Materials Synthesis and Processing, School of Materials Science and Engineering, Wuhan University of Technology, Wuhan 430070, China; lwh_803@whut.edu.cn (W.L.); shift@whut.edu.cn (T.S.); fang_liu3@whut.edu.cn (F.L.); 253632@whut.edu.cn (C.Y.); fanqiao@whut.edu.cn (F.Q.); hankang@whut.edu.cn (K.H.); jsmeng@whut.edu.cn (J.M.); 2 Hubei Longzhong Laboratory, Wuhan University of Technology (Xiangyang Demonstration Zone), Xiangyang 441000, China; 3 Department of Physical Science & Technology, School of Science, Wuhan University of Technology, Wuhan 430070, China

**Keywords:** short-chain sulfur, N-doped hollow carbon nanospheres, carbon composite anode, potassiation mechanism, potassium-ion battery

## Abstract

Carbon-based materials are one of the ideal negative electrode materials for potassium ion batteries. However, the limited active sites and sluggish diffusion ion kinetics still hinder its commercialization process. To address these problems, we design a novel carbon composite anode, by confining highly reactive short-chain sulfur molecules into nitrogen-doped hollow carbon nanospheres (termed SHC-450). The formation process involves the controlled synthesis of hollow polyaniline (PANI) nanospheres as precursors via an Ostwald ripening mechanism and subsequent sulfuration treatment. The high content of constrained short-chain sulfur molecules (20.94 wt%) and considerable N (7.15 wt%) ensure sufficient active sites for K^+^ storage in SHC-450. Accordingly, the SHC-450 electrode exhibits a high reversible capacity of 472.05 mAh g^−1^ at 0.1 A g^−1^ and good rate capability (172 mAh g^−1^ at 2 A g^−1^). Thermogravimetric analysis shows that SHC-450 has impressive thermal stability to withstand a high temperature of up to 640 °C. Ex situ spectroscopic characterizations reveal that the short-chain sulfur provides high capacity through reversible formation of K_2_S. Moreover, its special hollow structure not only provides ample space for highly active short-chain sulfur reactants but also effectively mitigates volume expansion during the sulfur conversion process. This work offers new perspectives on enhanced K^+^ storage performance from an interesting anode design and the space-limited domain principle.

## 1. Introduction

In recent years, lithium-ion batteries have become the main source of energy for many electric vehicles, communication devices and unmanned aerial vehicles due to their high energy density, quick charging and long cycle life [1]. However, the cost of lithium is increased by its scarcity and uneven distribution, which makes it unsuitable for widespread use in the field of large-scale energy storage [2]. Hence, potassium-ion batteries (PIBs) have gained interest due to nearly limitless supply of potassium resources and their physicochemical similarities to lithium [3]. Particularly, the low redox potential of potassium (−2.936 V (K^+^/K) vs. standard hydrogen electrode) is close to that of lithium (−3.040 V (Li^+^/Li)), implying comparable energy densities for potassium-ion and lithium-ion batteries [4,5,6]. Moreover, the weaker Lewis acidity of K^+^ compared to Li^+^ results in a smaller Stokes radius for solvated ions and faster ion transfer kinetics, indicating improved charge–discharge rate performance for PIBs [7,8]. However, the utilization of a high-capacity potassium metal anode encounters several obstacles, such as significant volume changes, uncontrollable dendrite growth, and unstable solid electrolyte interfaces, which compromise battery safety. The volume change can mechanically damage the fragile solid electrolyte interphase (SEI) in the repeated plating/stripping processes. As a result, non-uniform distribution of electric fields and K^+^ flux occur on the broken SEI, promoting deposition of potassium in irregular patterns and subsequent uncontrollable dendrite growth [9]. Furthermore, the exposed K dendrites with large surfaces continually react with the electrolyte, leading to a decrease in coulombic efficiency (CE) and the formation of inactive “dead K”. Accumulated dendrites can also pierce separators, causing short circuits, battery malfunction, and potential hazards such as fire and explosion [10]. Hence, the usage of potassium metal anodes as anode materials for commercial PIBs is challenging. There is an urgent and essential demand to explore suitable electrode materials that can overcome these issues and enable the development of high-performance PIBs.

Much work has gone into the development of potential anode materials [11]. Carbon materials are leading the way due to their environmental friendliness, high conductivity, design flexibility and commercial potential. However, the inherent limited capacity has hindered their use in high-energy PIBs [12]. To address this limitation, extensive efforts have been made to enhance their electrochemical performance, with structural engineering and heteroatom doping emerging as effective strategies [13]. In general, nanospheres with a hollow structure can benefit from reduced volume expansion, shorter diffusion routes, and a greater electrolyte–electrode contact area. Consequently, these materials demonstrate increased specific capacity, superior rate performance, and enhanced cycle stability [14]. Meanwhile, the doping of heteroatoms (B, N, O, P, S, etc.) has been proved to modulate electronic structure, widen interlayer distance, generate abundant active sites and improve the wettability with electrolyte [12]. Among the various heteroatom dopants, nitrogen doping is the most prevalent in carbon materials. Nitrogen-doped carbon typically consists of three distinct nitrogen-containing species: pyridinic-N, pyrrolic-N, and graphite-N. Pyrrole/pyridine-N has been found to decrease the adsorption energy of potassium ions, while graphite-N enhances electrical conductivity [15,16].

In addition to nitrogen doping, the doped S serves as electrochemically active sites by undergoing reversible reactions with K^+^. To date, a succession of hard carbon materials doped with sulfur have been synthesized for PIBs anodes through one-step carbonization of sulfur-containing precursors or chemical vapor deposition methods [17,18,19,20]. However, the specific capacity exhibited by these materials is still below 400 mAh g^−1^ [21]. The low doping level of hard carbon materials can be the reason for the limited improvement in performance, which comes from sulfur doping. Excessive sulfur doping can disrupt the conductive network of graphene layers and catalyze the decomposition of the electrolyte, resulting in low electronic conductivity and rapid capacity decay [22,23]. Goyenola et al. [24] used first principles calculations, effectively revealing the effects of incorporation of S in graphene-like model systems. Developing advanced materials for high-energy-density storage, conventional elemental sulfur (S_8_) also ranks among the top owing to its resource abundance and high theoretical capacity (1675 mAh g^−1^) [25]. Unfortunately, this elemental sulfur suffers from slow reaction kinetics and the dissolution of polysulfides, which result in a low sulfur utilization ratio, low coulombic efficiency and significant capacity degradation. In contrast, small sulfur molecules not only prevent the production of irreversible intermediates but also shorten the redox pathways, leading to superior reaction kinetics and reversibility [26]. However, the formation of K_2_S involves a much larger theoretical volume change, approximately 296%, compared to ~80% for Li_2_S [27]. This significant volume change makes creating anodes with high-capacity and long-term stability much more difficult. Lai et al. [28] used the synthetic growth concept model to accurately analyze the possible species resulting from the recombination of S fragments with atomic C. Therefore, integrating the customizable properties of carbon material structure and the high capacity of short-chain sulfur holds promise for developing a new high-capacity and stable anode for PIBs [29].

Herein, in order to solve the problem of low capacity and the terrible diffusion rate of carbon-based materials in the field of potassium-ion storage, we tailored high-electrochemical-activity short-chain sulfur molecules into nitrogen-doped hollow carbon nanospheres (denoted as SHC-450) to achieve high-capacity and fast potassium storage. N-doped hollow carbon nanospheres were synthesized by an Ostwald ripening process using aniline monomers as precursors and followed heat treatment. The investigation conducted by time-of-flight secondary ion mass spectrometry (TOF-SIMS) indicates that tiny sulfur molecules (S_x_, x ≤ 3) are confined to carbon nanopores or carbon defects. The hollow structure endows SHC-450 with extraordinary stability, while confined short-chain sulfur provides high capacity through fast and stable reaction with K^+^. Moreover, intrinsic N doping can introduce additional potassium adsorption sites to increase the specific capacity. Combining the above merits, SHC-450 exhibits a high specific capacity (463.28 mAh g^−1^ after 200 cycles at 0.1 A g^−1^), impressive cyclic stability (296.2 mAh g^−1^ after 2000 cycles at 1 A g^−1^) and superb rate capacity (172 mAh g^−1^ at 2 A g^−1^). Significantly, ex situ XPS revealed that short-chain sulfur provided high capacity through reversible breakage of C-S and S-S bonds. Moreover, ex situ HRTEM verified that K_2_S was the end product of covalent sulfur and S-S conversion. Thermogravimetric analysis showed that SHC-450 had impressive thermal stability to withstand a high temperature of up to 640 °C. Ex situ Raman analysis demonstrated long cycling stability mainly originating from reversible changes in the hollow sphere structure. This work demonstrates that introducing short-chain sulfur molecules in nitrogen-doped hollow carbon nanospheres can be an effective method to achieve high-capacity and stable potassium storage.

## 2. Materials and Methods

A typical method was employed to synthesize polyaniline (PANI) spheres. Volumes of 14 mL H_3_PO_4_, 5.10 g H_2_O_2_, 2.08 g aniline (ANI) monomer and 0.074 g FeCl_3_ were dissolved in 450 mL deionized water with vigorous stirring at room temperature for 30 min. Then, the homogeneous mixture was transferred into a 500 mL Teflon-lined autoclave and heated at 140 °C for 6 h [30]. After cooling to room temperature, the PANI spheres were washed by deionized water and ethanol in turn for three times, and then dried overnight at 70 °C in oven. The SHC-450 samples were prepared by mixing PANI spheres with sulfur (mass ratio is 1:3) and then pyrolyzed at 450 °C for 5 h under slow Argon flow. The heating rate is 5 °C min^−1^. The HC-450 samples were prepared by directly carbonizing the PANI spheres at 450 °C for 5 h under argon flow.

## 3. Results

### 3.1. Schematic Diagram of the SHC-450 Synthesis Process and Principle of Hollow Sphere Structure Formation

**Figure 1:**
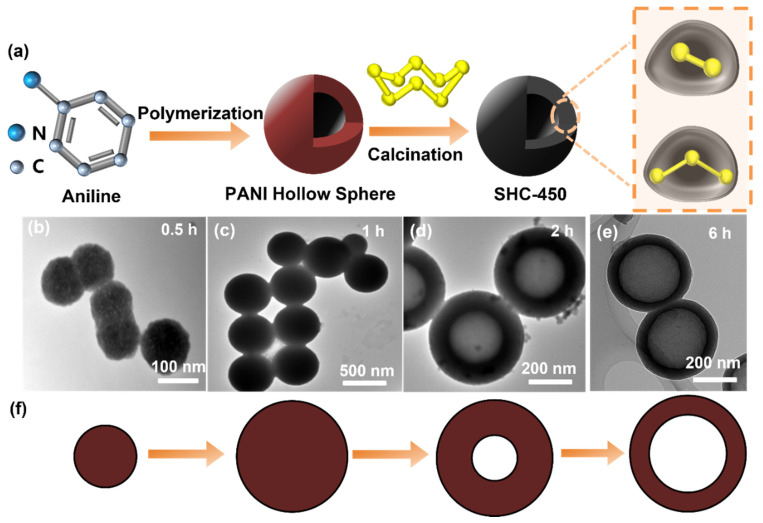
(**a**) Schematic illustration of the synthesis process for SHC-450. (**b**–**e**) TEM images of PANI at different reaction times from 0.5 to 6 h at the same reaction temperature of 140 °C. (**f**) The diagram of structure evolution for hollow PANI nanospheres.

Figure 1a shows a prototypical synthesis procedure for SHC-450. Initially, hollow polyaniline (PANI) spheres are fabricated through intermolecular polymerization of aniline monomers within an aqueous medium. This synthesis process employs hydrogen peroxide as an oxidative catalyst and a trace of ferric chloride as an initiator. Subsequently, the hollow PANI spheres and powdered sulfur are amalgamated via manual grinding and then subjected to controlled heating under an argon atmosphere to yield the SHC-450 composite. At temperatures surpassing 115 °C, sulfur undergoes a transition into a liquid state, and as the temperature ascends to 450 °C, cyclo-S_8_ molecules disintegrate into radicals. By virtue of the melting–diffusion mechanism, short-chain sulfur can enter the pore space. Upon the gradual cooling of the system, the liquid sulfur congeals, effectively entrapping these chain-like sulfur molecules within the micropores, preventing their reversion to ring structures (S_8_) at room temperature [31].

We subsequently investigated the formation process of the hollow PANI nanospheres. The evolution of uniform hollow PANI spheres is elucidated through transmission electron microscopy (TEM) observations. PANI was synthesized at a constant reaction temperature of 140 °C for varying durations. As illustrated in Figure 1b–e, within 0.5 h of reaction time, the reactants swiftly nucleate, generating nuclei with a diameter of approximately 100 nm, which then undergo rapid growth, reaching a size of 400 nm after 1 h. Extending the reaction time to 2 h yields spheres of almost identical size, albeit with a transformation in morphology from solid spheres to yolk–shell structures, as revealed in the TEM image (Figure 1d). Hollow nanospheres with delicate shells are produced when the reaction time is further extended to 6 h (Figure 1e). This observed transformation can be expounded upon through the lens of an inside-out Ostwald ripening mechanism (Figure 1f). During the nucleation stage, the inner microcrystals are comparatively diminutive and feature elevated surface energy with a heightened solubility form. In contrast, the outer surface crystals, originating in later stages, are more substantial and thus more thermodynamically stable. Consequently, the central region of the sphere undergoes dissolution and reformation on the spherical shell, leading to the development of a hollow cavity and, in turn, the creation of the hollow structure [32].

### 3.2. Morphology and Structural Characterization

**Figure 2:**
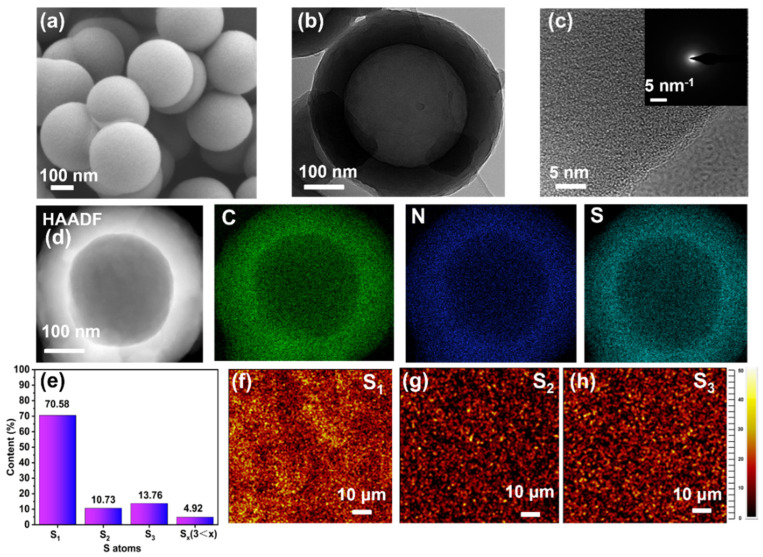
Characterization of SHC-450. (**a**) SEM image, (**b**,**c**) TEM images at different magnifications of SHC-450. Inset of (**c**) is an SAED pattern. (**d**) C, N, and S element mapping images of SHC-450. (**e**) Contents of sulfur species of SHC-450 calculated based on the result of TOF-SIMS. (**f**–**h**) Intensity mapping of S_1_, S_2_, and S_3_.

The TEM and SEM images reveal that SHC-450 exhibits a consistently spherical morphology, with a diameter of approximately 400 nm and sheet thickness of 40 nm (Figure 2a,b). The selected area electron diffraction (SAED) pattern indicates a highly disordered structure without a visible crystalline order in SHC-450 (Figure 2c), implying the absence of S_8_ molecules within the structure. Furthermore, the TEM elemental mapping images of SHC-450 (Figure 2d) demonstrates the homogenous distribution of the C, N, and S elements in the sample. Through the same structural characterizations, the HC-450 is observed to have a comparable hollow morphology without sulfur element (Figure S1). The TOF-SIMS technique was employed to investigate the molecular status of sulfur, which verified the several states of microporous-confined sulfur present in SHC-450.

Figure S2 displays the negative ion polarity data that were gathered during the TOF-SIMS investigation. The relative mass intensities calculated based on the anion shown in Figure 2e reveals that the predominant sulfur species is single-atom sulfur (70.58%), followed by S_3_ (13.76%) and S_2_ (10.73%) with reduced intensities. Notably, trace amounts of large sulfur molecules (4.9% in total for S_4_–S_7_) are also detected. In addition, Figure 2f–h show intensity mapping of S_1_, S_2_ and S_3_, which confirms the uniform distribution of various forms of sulfur in SHC-450. Overall, encapsulated sulfur primarily exists in the form of S_2_–S_3_, and there is minimal presence of long-chain sulfur species within the sample.

**Figure 3:**
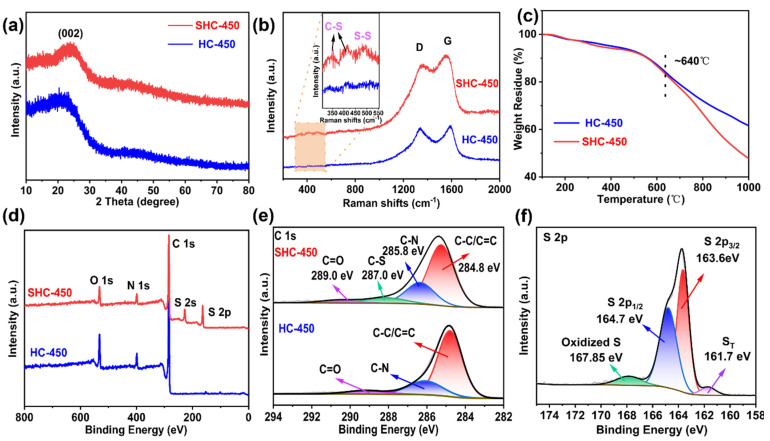
(**a**) XRD pattern, (**b**) Raman spectra, (**c**) TGA curve, (**d**) High-resolution XPS patterns of SHC-450 and HC-450. (**e**) Deconvolution C 1s spectrum; (**f**) Deconvolution S 2p spectrum.

The XRD analysis of SHC-450 reveals a single dispersive peak at approximately 23.8° without other miscellaneous peaks (Figure 3a). The unique peak corresponds to the (002) lattice planes of the hard carbon, indicating no cyclo-S_8_ remained [33]. Raman spectra is used to further confirm the type of bonds created by sulfuration treatment. As exhibited in Figure 3b, there are distinct peaks at approximately 335 and 410 cm^−1^, which are attributed to covalent C-S bonding. In addition, the characteristic peak at 487 cm^−1^ represents a S-S bond [34]. As shown in Figure 3c, the SHC-450 sample demonstrates remarkable thermal stability, which was measured by thermogravimetric (TGA). Appreciable weight loss in SHC-450 does not begin to occur until approximately 640 °C. This temperature threshold is significantly higher than 300 °C since beyond this point, pristine cyclo-S_8_ evaporation takes place [35]. The improved thermal stability for SHC-450 can be accredited to the robust containment provided by the microporous carbon host as well as the formation of robust covalent bonds between the S and C atoms [36]. The CHNSO elemental analyzer is used to determine the mass fraction of elements S and N. The results (Table S1) show that SHC-450 is rich in nitrogen, sulfur, and oxygen elements, with a N content of 7.15 wt%, S content of 20.94 wt%, and an O content of 12.93 wt%. The N_2_ adsorption–desorption isotherms of SHC-450 and the sulfur-free directly carbonized HC-450 are shown in Figure S3. As a result, SHC-450 and HC have BET surfaces and pore volume of 12.75 m^2^ g^−1^, 248.57 m^2^ g^−1^ and 0.024 cm^3^ g^−1^,0.156 cm^3^ g^−1^, respectively. The specific surface and pore volume of SHC-450 are slightly decreased can be attributed to the filling of nano-scale sulfur molecules into the nanopores, which restricts the infiltration of nitrogen [37].

X-ray photoelectron spectroscopy (XPS) spectra is employed to examine the elemental chemical states of both SHC-450 and HC-450 materials. SHC-450 exhibits the unique peaks associated with S 2p and S 2s, suggesting that sulfur species have been successfully introduced. The high-resolution C 1s profile of SHC-450 can be deconvoluted into the four peaks at 284.8 eV, 285.8 eV, 287eV, and 289 eV, corresponding to C-C/C=C, C-N, C-S and C=O, respectively. However, only three peaks, C-C/C=C (284.7 eV), C-N (286 eV), and O-C=O (288.5 eV) are observed in the XPS spectra of HC-450 (Figure 3e) [38]. As shown in Figure 3f, the high-resolution S 2p spectrum of SHC-450 is deconvolved into four peaks: C-S (163.6 eV), S-S (164.7 eV), oxidized S (167.85 eV) and terminal sulfur species S_T_ (161.7 eV) [39].

### 3.3. Electrochemical Measurements

**Figure 4:**
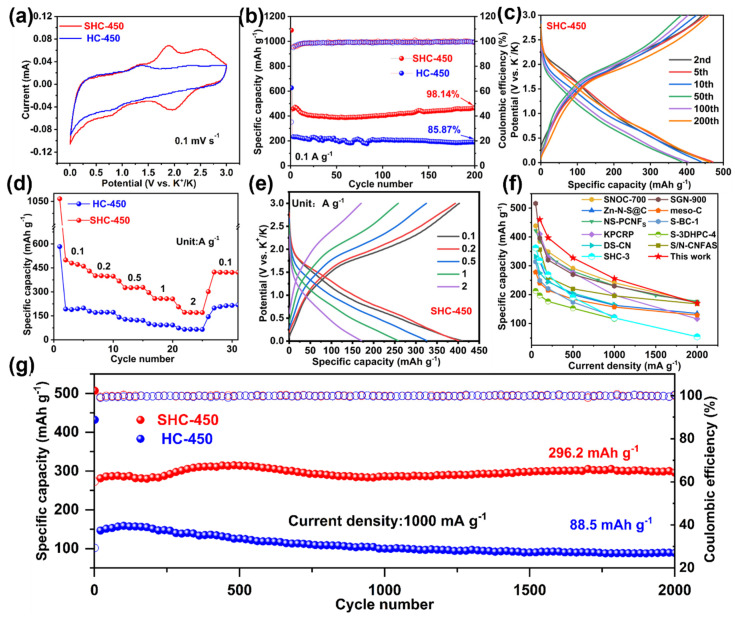
Electrochemical performance test of SHC-450 and HC-450 electrodes for a PIB anode. (**a**) The CV curves at a scan rate of 0.1 mV s^−1^. (**b**) Cycling performance of SHC-450 and HC-450 tested at a current density of 0.1 A g^−1^. (**c**) Charge–discharge profiles for SHC-450 during cycling. (**d**) Rate performance of SHC-450 and HC-450 at various current densities from 0.1 to 2 A g^−1^. (**e**) The corresponding charge and discharge voltage profiles of SHC-450 at different current densities. (**f**) Rate performance compared to other reported heteroatom-doped carbonaceous materials. (**g**) Cycling performance of SHC-450 and HC-450 tested at a current density of 1.0 A g^−1^.

To evaluate the significance of tailoring sulfur species into nitrogen-doped hollow carbon nanospheres, the potassium storage performance of two samples is compared comprehensively. The electrochemical reaction process is verified using cyclic voltammetry (CV) at 0.1 mV s^−1^ in the potential range from 0.01 to 3 V. SHC-450 exhibits significantly greater area than the HC-450 at the second cycle, indicating its high electrochemical activity and superior reaction kinetics (Figure 4a). In the case of SHC-450, the clear and sharp cathodic peak observed at approximately 1.1 V during the initial cycle originates from the creation of a solid electrolyte interphase (SEI), which subsequently diminishes in subsequent cycles [40]. As shown in Figure S4, a thin SEI layer is observed by HRTEM after discharging to 0.01 V at the initial cycle. At 0.7/1.8 V and 1.9/2.5 V, two pairs of revertible peaks are observed, suggesting that there are at least two distinct reduction reactions of the multi-step potassiation process that result from the potassiation of sulfur with different atomic environments [41] and chain lengths [42]. In the following three CV cycles, curves overlap well, indicating the good cycle stability and reversibility characteristic of the SHC-450 electrode (Figure S5a). By contrast, Figure S5b displays the first four cycles of CV curves of HC-450. The redox peaks at 0.01/1.3 V are ascribed to the intercalation/deintercalation of K^+^. The weaker and broadened peaks at 0.7 V and 2.3 V suggest the interaction between potassium and nitrogen species [43].

The cycle capabilities of the two samples are evaluated through testing at a current density of 0.1 A g^−1^. The cycling capacities and corresponding coulombic efficiency are shown in Figure 4b. After 200 cycles, the SHC-450 sample delivers a high reversible capacity of 463.2 mAh g^−1^ and a high-capacity retention of 98.14%, which are higher than those of HC-450 (low capacity of 220 mAh g^−1^ and only 85.87% capacity retention). Figure 4c and FigureS 6a illustrate the galvanostatic charge–discharge profiles of SHC-450 and HC-450 at a current density of 0.1 A g^−1^ from the 2nd to the 200th cycle. As can be seen, SHC-450 exhibits a clearly sloped charge platform at approximately 1.8 V in comparison to HC-450, which conforms to CV analysis, further showing the redox interaction between potassium and short-chain sulfur with slight changes among the various curves. The TEM image of SHC-450 after 200 cycles at 0.1 A g^−1^ is displayed in Figure S7, and the well-maintained hollow nanosphere structure confirms its long cycling stability. The initial discharge–charge-specific capacities are 1089/491 and 626/220 mAh g^−1^ at 0.1 A g^−1^ for SHC-450 and HC-450 electrodes (Figure S8), with a corresponding ICE of 45.1% and 35.1%, respectively. The improved initial coulombic efficiency of SHC-450 can be related to lower specific surface areas, which means less formation of SEI [44]. Moreover, the corresponding TEM images and elemental mapping are shown in Figure S9, which confirms the distribution of elements C, N, S, and K.

The rate performance is also evaluated at different rates from 0.1 to 2 A g^−1^ (Figure 4d). At 0.1, 0.2, 0.5, 1 and 2 A g^−1^, SHC-450 delivers average discharge capacities of 477, 399, 326, 257 and 172 mAh g^−1^, while the corresponding reversible capacities of HC-450 are 193, 171, 124, 92, and 65 mAh g^−1^, respectively. For SHC-450, when the current density returns to 0.1 A g^−1^, it exhibits a recovery to a capacity of 422 mAh g^−1^, suggesting exceptional rate performance and extraordinary stability. The corresponding charge–discharge curves are shown in Figure 4e and Figure S6b. SHC-450 preserves voltage profiles well even at a higher current density, which provides further confirmation of the outstanding rate performance. Importantly, the rate performance of the SHC-450 electrode outperforms that of most previously reported carbonaceous anodes (Figure 4f).

The electrodes are subjected to long-term cycling tests at a high current density of 1 A g^−1^ to further evaluate the cycling stability of electrodes. Figure 4g shows the long cycling performance of SHC-450 and HC-450 over 2000 cycles. Impressively, with a CE of approximately 100%, SHC-450 can provide 296.2 mAh g^−1^ reversible capacity without any decay, which is much higher than that of HC-450 (88.5 mAh g^−1^, 70.3% retention over 2000 cycles). This further demonstrates that incorporating fragmented sulfur into microporous carbon may be an effective approach to creating a high-capacity, ultra-stable electrode material. In order to further investigate the kinetics of K^+^ diffusion kinetics during the charge–discharge process, a galvanostatic intermittent titration technique (GITT) is used (Figure S10a), and the calculation details can be found in Figure S10b,c. Impressively, the D-value of SHC-450 surpasses HC-450 at the most potentials, indicating its enhanced reaction kinetics and good rate performance.

## 4. Discussion

**Figure 5:**
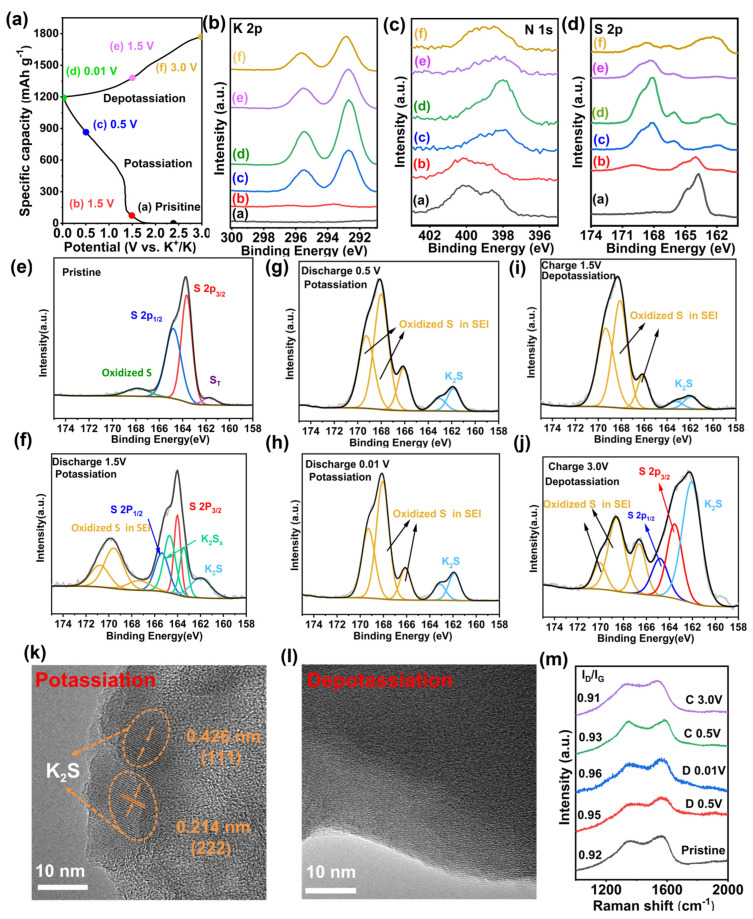
Electrochemical reaction of SHC-450 for K^+^ storage. (**a**) The initial charge–discharge profiles at 0.1 A g^−1^ marked point for XPS testing. (**b**) K 2p, (**c**) N 1s, and (**d**) S 2p. (**e**–**j**) Fitting of the S 2p XPS spectra of the SHC-450 electrode under different states of charge and discharge which are marked in (**a**). (**k**,**l**) HRTEM images of SHC-450 potassiation to 0.01 V and depotassiation to 3.0 V. (**m**) Ex situ Raman analysis of SHC-450 at different states.

To gain a comprehensive understandings of the (de)potassiation mechanism in SHC-450, ex situ XPS, Raman and TEM analyses are applied at different stages during the initial cycle. The selected potentials for the XPS test are shown in Figure 5a. For K 2p spectra in Figure 5b, two peaks at approximately 292.5 and 295.5 eV shift to a lower binding energy with increasing overall intensity during discharge, implying the creation of K-C, K-S and K-N bonds in SHC-450 (Figure S11) [45]. During the charge process, two peaks decrease in intensity and come back to a higher binding energy. However, even at 3.0 V, two peaks subsisted due to some irreversible reactions like irreversible trapping of K in SEI [46]. In addition, the XPS N 1s spectra are shown in Figure 5c and reveal changes in N during the (de)potassiation process. When pristine, four peaks at 398.5, 400.1, 401.6 and 404.4 eV composed of pyridinic nitrogen (N-6), pyrrolic nitrogen (N-5), graphitic nitrogen (N-Q) and oxidized nitrogen (N-O) are observed in Figure S12. The binding energy of nitrogen species shifts to lower values during discharge, followed by a subsequent increase during the charging process, indicating that the electron transforms from K atom to the N-dopants during potassiation and N doping sites release both K^+^ and charge in the depotassiation state. This observation also indicates that N doping can provide reversible K^+^ storage sites to improve the specific capacity [47].

To explain the mechanism of the reaction between sulfur species and potassium, sulfur element is also detected by XPS. These results are shown in Figure 5d–j. For the pristine SHC-450, four distinct peaks are observed at 163.6 eV, 164.7 eV, 167.85 eV, and 161.7 eV, which correspond to the peaks of C-S, S-S, oxidized S and S_T_ (Figure 5e). When discharge to 1.5 V, the presence of S 2p is still evident, along with the emergence of sulfide (K_2_S_X_) peaks at 164.5/163.4 eV, which can be attributed to the initial reaction of K^+^ with the bonding of -S-S- [22]. An additional peak at 161.97 eV is related to the formation of sulfides (K_2_S), which also confirms that there are at least two distinct reduction reactions of the potassiation process that result from sulfur species with different atomic environments and chain lengths.

Moreover, the S 2p spectra in Figure 5f indicates three peaks with a binding energy exceeding 164 eV. These peaks are attributed to the oxidized S originating from the KFSI-derived solid electrolyte interphase [21]. Further into the potassiation process, the intensity of two obvious peaks at 163.08 eV and 161.85 eV belonging to K_2_S increases at the expense of S 2p, which disappears, implying that the C-S and S-S bonds react with K^+^. Furthermore, the intensity of the three peaks above 164 eV experiences a swift growth, suggesting the SEI is generated rapidly (Figure 5g) [41]. After potassiation by discharging to 0.01 V, except for the increases in the peak intensity of K_2_S, there is no significant change. Following depotassiation (Figure 5g,i), the intensity of the oxidized S substance gradually decreases, while S 2p remerges and the intensity gradually increases. Upon complete depotassiation to 3 V, signals attributed to C-S and S-S bonds can be detected, suggesting the recovery of short-chain sulfur in SHC-450. Some residual K_2_S is still observed, which is due to the irreversibility of sulfate remaining in SEI [48].

Ex situ HRTEM is employed to analyze SHC-450 after potassiation to 0.01 V and subsequent depotassiation to 3.0 V. As shown in Figure 5k, the observed lattice spacings of 0.214 nm and 0.426 nm correspond to the (222) and (111) planes in K_2_S. The HRTEM image of the charged state is displayed in Figure 5l, which shows an amorphous structure once more. This finding suggests that the conversion of short-chain sulfur to discharge products of K_2_S is reversible, which is in agreement with the above results [49].

Ex situ Raman spectra is also collected to further confirm the changes in the structure. The increase in the ID/IG values from 0.92 to 0.96 upon discharging to 0.01 V primarily reflects the increase in disorder within the carbonaceous structure, attributed to the continuous embedding of K^+^ and the conversion of short-chain sulfur. During the subsequent depotassiation process, with a voltage from 0.01 to 3.0 V, the ID/IG ratio reverts to its initial state, indicating the reversibility of structural change during the potassiation/depotassiation process [50,51].

## 5. Conclusions

To solve the problems of the large volume expansion, low capacity and poor diffusion dynamics of carbon materials in the field of potassium-ion batteries. We innovatively designed and synthesized a high proportion of 20.94 wt% short-chain sulfur embedded into nitrogen-doped hollow carbon to achieve high capacity. Highly chemically active short-chain sulfur and nitrogen doping can provide many active sites. Nano hollow sphere structure can alleviate volume expansion and shorten K^+^ diffusion path to achieve stable and fast potassium storage. The formation mechanism is clearly revealed, involving an Ostwald ripening mechanism and subsequent sulfuration treatment. The short-chain sulfur (S_x_, x ≤ 3) is validated by TOF-SIMS. Ex situ XPS and HRTEM reveal that the short-chain sulfur exhibits high reactivity towards K^+^, which leads to the reversible cleavage of C-S and S-S bonds, ultimately resulting in the formation of K_2_S as the final product. Thermogravimetric analysis shows that SHC-450 has impressive thermal stability to withstand a high temperature of up to 640 °C. Ex situ Raman reveals the reversible structural changes during the charging/discharging process, indicating that the special hollow structure endows SHC-450 with extraordinary stability by effectively mitigating volume expansion during the sulfur conversion process. As a result, SHC-450 exhibits a high specific capacity (463.28 mAh g^−1^ after 200 cycles at 0.1 A g^−1^) and a superior longer cycle life (capacity of 296.2 mAh g^−1^ can be maintained after 2000 cycles at 1 A g^−1^). This work provides a new avenue to develop novel anode materials for KIBs.

## Data Availability

Data are available upon request.

## Supplementary Materials

The following supporting information can be downloaded at: https://www.mdpi.com/article/10.3390/nano14060550/s1, Figure S1: SEM, TEM, and EDS images of HC-450; Figure S2: TOF-SIM mass spectra of the SHC-450 sample; Figure S3: Nitrogen adsorption−desorption isothermal curves of SHC-450 and HC-450; Figure S4: TEM image of SHC-450 discharge to 0.01 V at the initial cycle; Figure S5: TEM image of SHC-450 discharge to 0.01 V at the initial cycle; Figure S6: Charge–discharge profiles for HC-450; Figure S7: SEM and TEM images of SHC-450 after 200 cycles at 0.1 A g^−1^; Figure S8: The charge–discharge profile and specific capacity of SHC-450 and HC-450 for the initial cycle; Figure S9: EDS mapping of the potassiated SHC-450; Figure S10: GITT profiles and corresponding K^+^ diffusion coefficients of SHC-450 and HC-450; Figure S11: K 2p XPS spectra of SHC-450 when discharging to 0.01 V. Figure S12: N 1s XPS spectra of SHC-450; Table S1: A comparison of element contents of SHC-450 and HC-450 based on COHNS analysis.
