# Supplementary material for: Short-Chain Sulfur Confined into Nitrogen-Doped Hollow Carbon Nanospheres for High-Capacity Potassium Storage
Source: Nanomaterials (Basel). 2024 Mar 20;14(6):550. doi: 10.3390/nano14060550 (PMC10976248; doi:10.3390/nano14060550)
Supplement: Supplementary file 1 [file nanomaterials-14-00550-s001.zip › nanomaterials-2921185-supplementary.pdf]

Supporting information

Short-Chain Sulfur Confined into Nitrogen-Doped Hollow Carbon Nanospheres for High-Capacity Potassium Storage

Wenhan Liu [1], Tengfei Shi [1], Fang Liu [1], Chen Yang [1], Fan Qiao [1], Kang Han [1], Chunhua Han [1,2,*], Jiashen Meng [1] and Xuanpeng Wang [1,2,3,*]

[1] State Key Laboratory of Advanced Technology for Materials Synthesis and Processing, School of Materials Science and Engineering, Wuhan University of Technology, Wuhan 430070, China;
lwh_803@whut.edu.cn (W.L.); shift@whut.edu.cn (T.S.);
fang_liu3@whut.edu.cn (F.L.);
253632@whut.edu.cn (C.Y.); fanqiao@whut.edu.cn (F.Q.);
hankang@whut.edu.cn (K.H.); jsmeng@whut.edu.cn (J.M.)

[2] Hubei Longzhong Laboratory, Wuhan University of Technology (Xiangyang Demonstration Zone),
Xiangyang 441000, China

[3] Department of Physical Science & Technology, School of Science, Wuhan University of Technology,
Wuhan 430070, China

* Correspondence: hch5927@whut.edu.cn (C.H.); wxp122525691@whut.edu.cn (X.W.);
Tel.: +86-13545284506 (X.W.)

Material characterizations

JSM-7100F scanning electron microscope (SEM) and Titan Themis G2 60-300 transmission electron microscopy (TEM) were utilized to characterize the microscopic features of the samples. Additionally, the TEM is equipped with an X-ray energy spectrum analysis system, which aids in the qualitative analysis of material composition and distribution. The D8 Advance powder X-ray diffractometer was used to determine the structures of the as-prepared materials with Cu Kα. The corresponding voltage and current parameters are 20 kV and 10 A, respectively, and the test angle is 10–80° at a scan rate of 5° min^{-1}. TGA was performed on an STA-449F5 instrument in Argon atmosphere with a temperature

ramp of 10 °C min^{-1} to verify the thermal stability of SHC-450. Raman spectroscopy experiments were performed on the HORIBA LabRAM HR Evolution micro-Raman spectroscopy system with the 532 nm laser. The valence states investigation was conducted using X-ray photoelectron spectroscopy (XPS) with a VG Multilab 2000 system. To mitigate the interference caused by potassium salts in the electrolyte, the electrodes were retrieved by disassembling the cells in a glovebox, and any remaining salt residue was eliminated using DME solvent. The BET surface area was calculated from nitrogen adsorption isotherms collected at 77 K using a Tristar 3020 instrument. Time-of-flight secondary ion mass spectrometry (TOF-SIMS) was conducted on PHI nano-TOF III.

Electrochemical measurements

The SHC-450 and HC-450 electrodes were prepared by casting a homogeneous slurry, which was achieved by blending the active material, Ketjen Black, and binder (sodium carboxymethyl cellulose/Styrene-Butadiene Rubber, weight ratio of 1:1) in deionized water at a weight ratio of 7:2:1 onto a Cu foil and then dried at 70°C for 10h. Subsequently, circular sections with a diameter of 10 mm were excised from these desiccated electrodes, resulting in a mass loading of 0.8–1 mg cm^{-2} of active material. The 2016 coin cells were assembled in a pure argon gas filled glovebox with both oxygen and water below 0.01 ppm. A potassium foil was used as the counter electrode. Grade GF/D Whatman glass microfiber filter was employed as the separator and 5 M KFSI in 1,2−dimethoxyethane (DME) was used as the electrolyte. Galvanostatic charge–discharge measurements were performed using a multichannel battery testing system (LAND CT2001A) in the potential range from 0.01 to 3.0 V (vs. K/K^+) at different current densities. CVs were collected at room temperature using an Autolab PGSTAT302N. A LAND CT2001A multichannel testing system was used to measure the GITT curve as well. It discharged at a rate of 50 mA g^{-1} for

10 minutes before relaxing in an open circuit for 30 minutes. The value of D_{K+} can be calculated in below Equation

$$D = \frac{4}{\pi\tau}\left(\frac{m_B V_M}{M_B S}\right)^2\left(\frac{\Delta E_S}{\Delta E_\tau}\right)^2$$

where τ is the duration of the current impulse; m_B, S, V_M, and M_B denote the mass load of the electrode material, active electrode surface area, molar volume and molar mass of carbon, respectively. ΔEs is the quasi-thermodynamic equilibrium potential difference before and after the current pulse, and ΔEτ represents the potential difference during the current pulse.

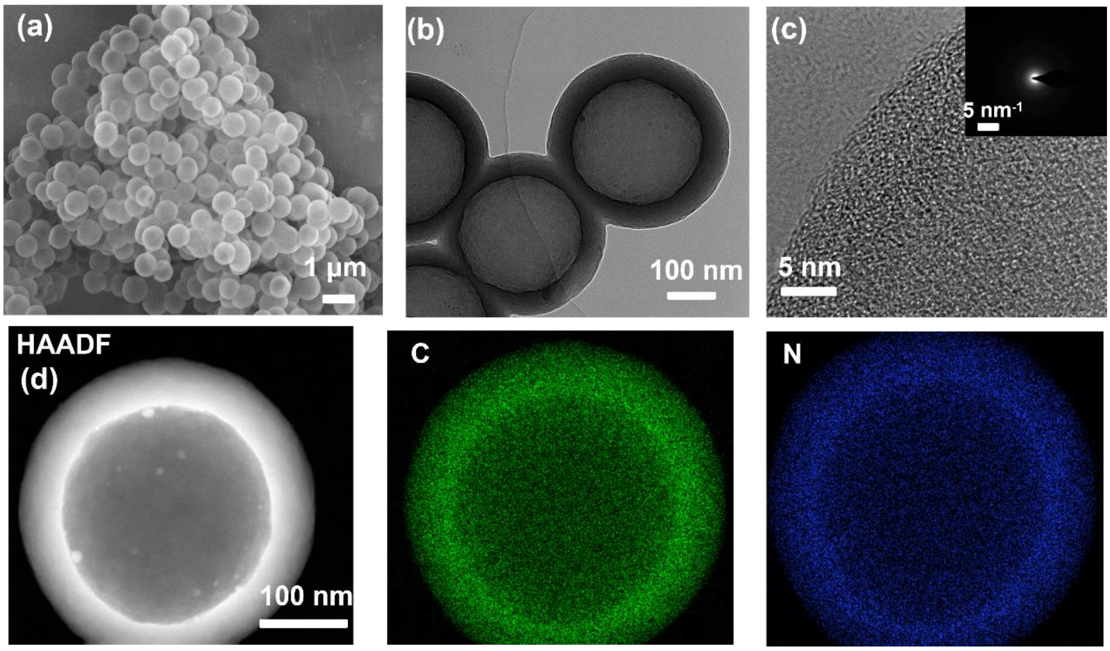

Figure S1. Characterization of HC-450. (a) SEM image. (b–c) TEM images at different magnifications of HC-450. Inset of (c) is an SAED pattern; (d) C and N element mapping images of HC-450.

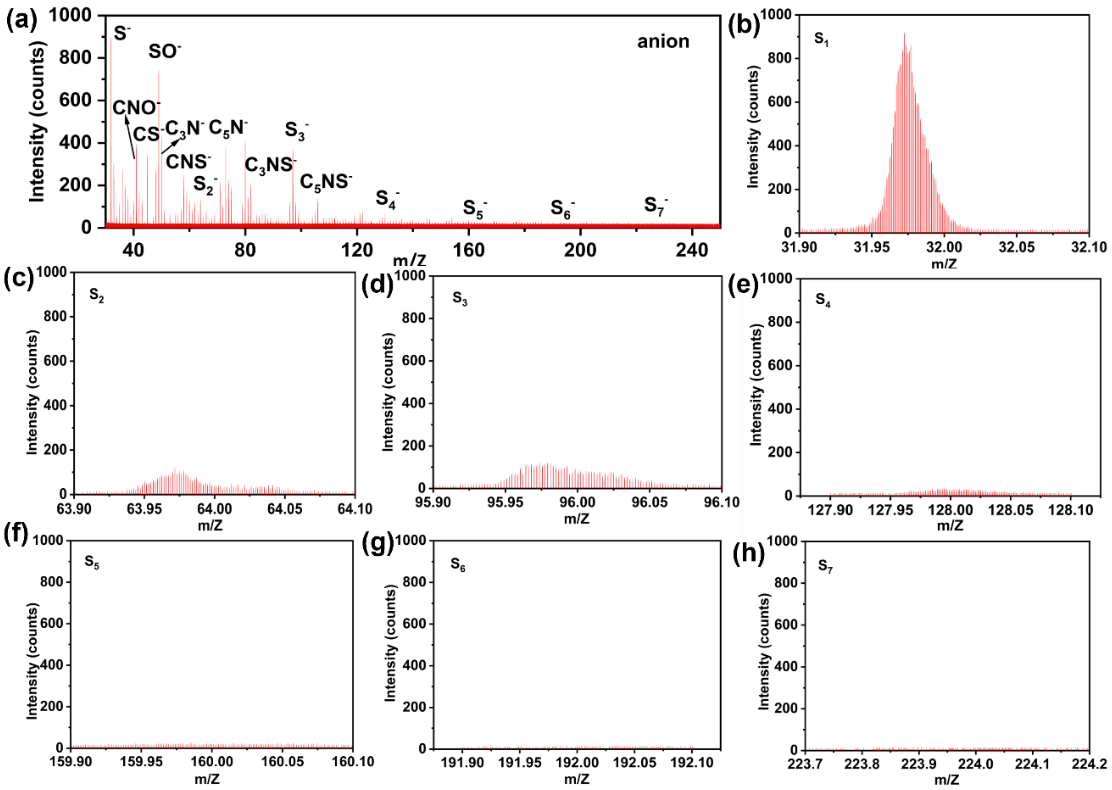

Figure S2. TOF-SIM mass spectrums of the SHC-450 sample.

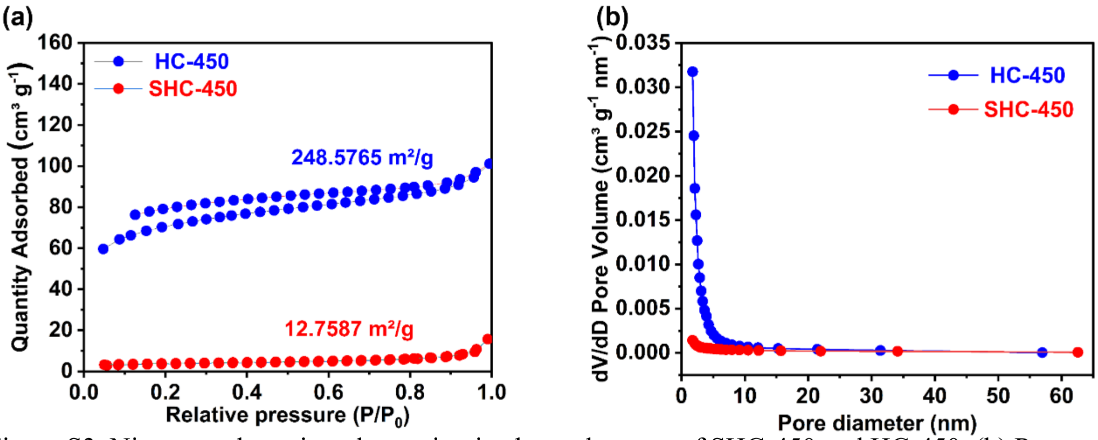

Figure S3. Nitrogen adsorption–desorption isothermal curves of SHC-450 and HC-450. (b) Pore size distribution of SHC-450 and HC-450.

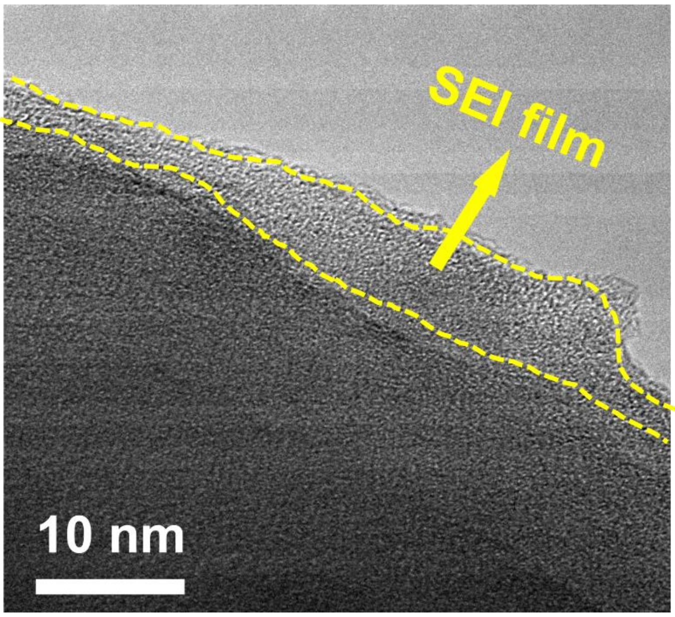

Figure S4. High-resolution TEM image of SHC-450 discharge to 0.01 V at the initial cycle.

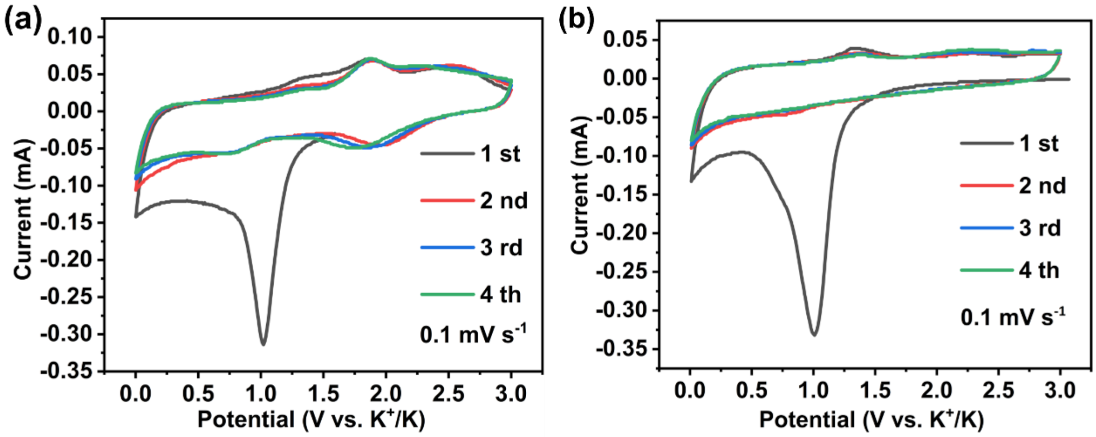

Figure S5. CV curves of SHC-450 and HC-450 at a scan rate of 0.1 mV s^{-1}

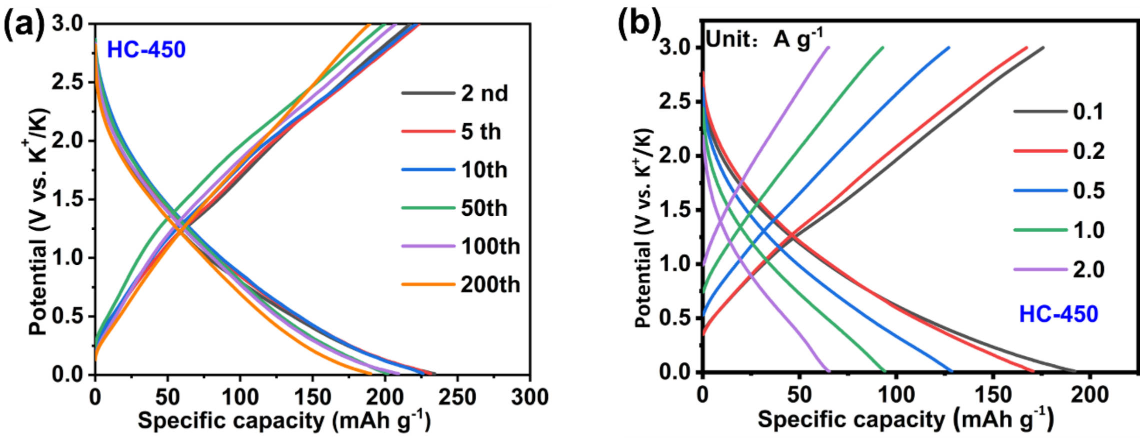

Figure S6. (a) Charge–discharge profiles for HC-450 during cycling at current density of 0.1 A g^{-1}. (b) The corresponding charge and discharge voltage profiles of HC-450 at current densities from 0.1 to 2 A g^{-1}.

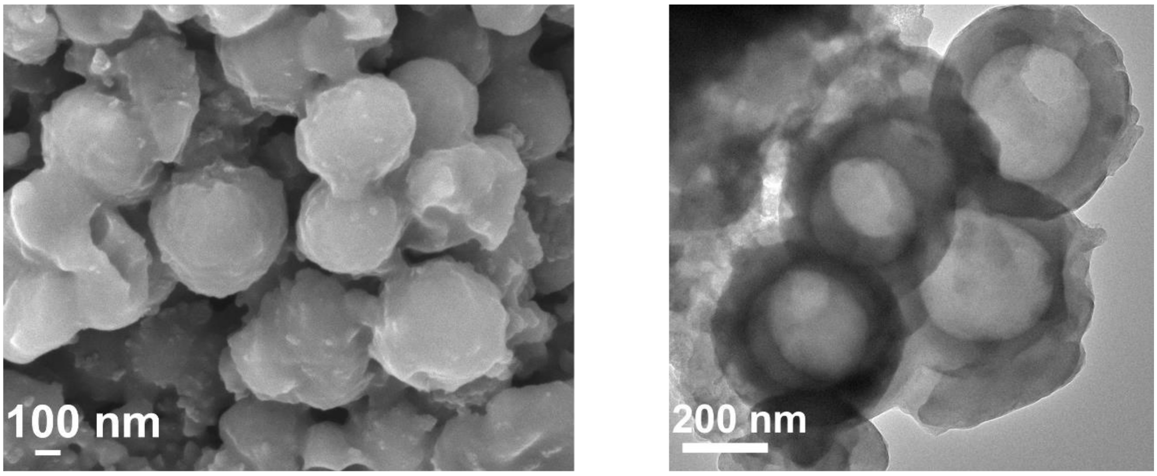

Figure S7. High-resolution SEM and TEM images of SHC-450 after 200 cycles at 0.1 A g^{-1}.

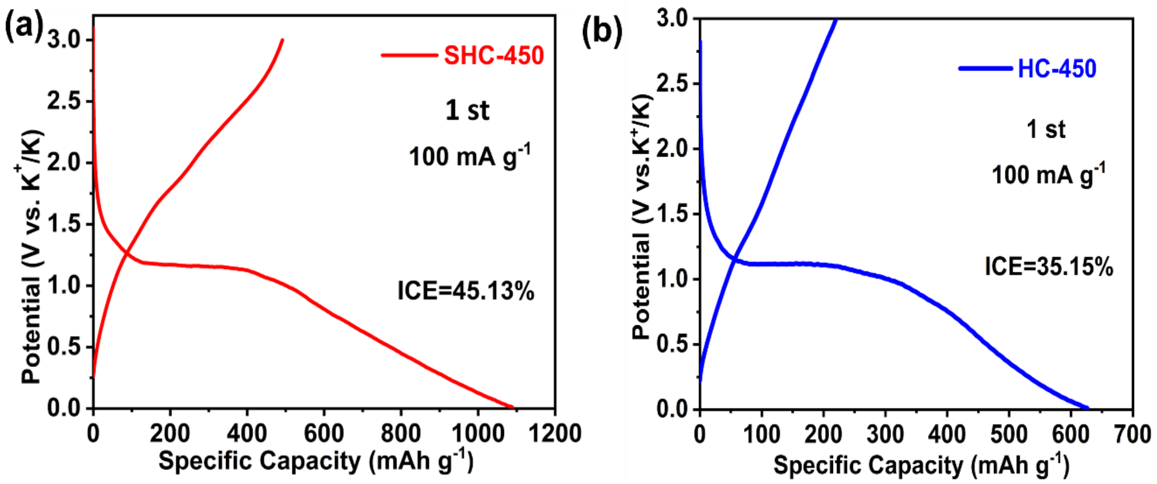

Figure S8. The charge–discharge profile and specific capacity for the initial cycle: (a) SHC-450 and (b) HC-450.

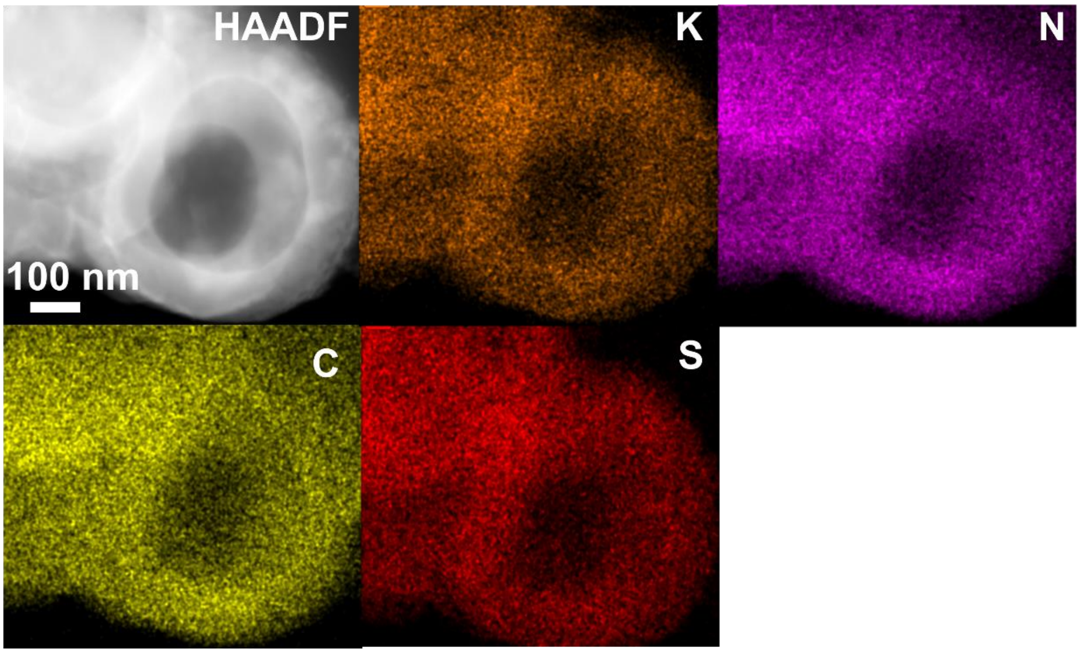

Figure S9. EDS mapping of the potassiated SHC-450.

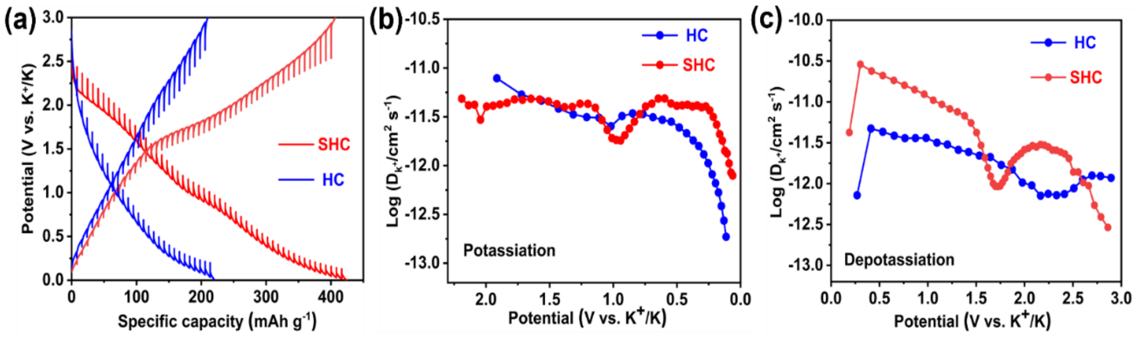

Figure S10. GITT profiles and corresponding K^+ diffusion coefficients of SHC-450 and HC-450 after the five cycles at 0.1 A g^{-1}.

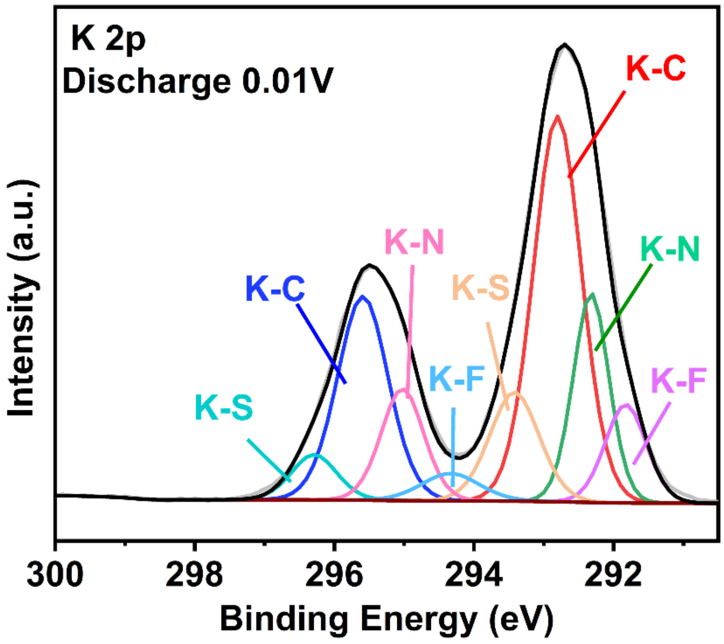

Figure S11. K 2p XPS spectra of SHC-450 when discharging to 0.01 V.

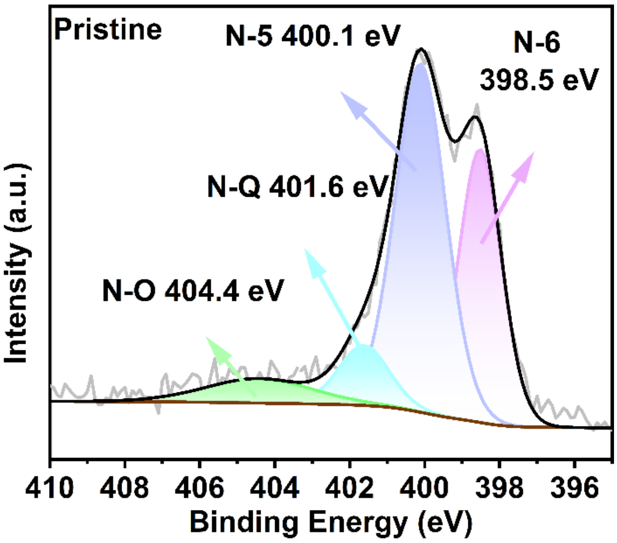

Figure S12. N 1s XPS spectra of SHC-450.

Table S1. A comparison of element contents of SHC-450 and HC-450 based on COHNS analysis.

	**Mass Fraction (wt%)**				
**Samples**	**C**	**H**	**O**	**N**	**S**
SHC-450	55.15	2.108	12.931	7.15	20.948
HC-450	72	3.279	14.616	8.55	0.583
